# Sex Differences in Cancer-Associated Thrombosis

**DOI:** 10.3390/ijms27052515

**Published:** 2026-03-09

**Authors:** Andrea Giachi, Davide Santagata, Addolorata Truma, Andrea Artoni, Paolo Bucciarelli, Luca Valenti, Cihan Ay, Roberta Gualtierotti

**Affiliations:** 1 Angelo Bianchi Bonomi Hemophilia and Thrombosis Center, Fondazione IRCCS Ca’ Granda Ospedale Maggiore Policlinico, 20122 Milan, Italy; andrea.giachi@policlinico.mi.it (A.G.); addolorata.truma@policlinico.mi.it (A.T.); andrea.artoni@policlinico.mi.it (A.A.); paolo.bucciarelli@policlinico.mi.it (P.B.); 2 Department of Medicine and Surgery, Research Center on Thromboembolic Disorders and Antithrombotic Therapies, University of Insubria, 21100 Varese, Italy; dsantagata@studenti.uninsubria.it; 3 Department of Pathophysiology and Transplantation, Università degli Studi di Milano, 20122 Milan, Italy; luca.valenti@unimi.it; 4 Precision Medicine, Biological Resource Center and Transfusion Medicine, Fondazione IRCCS Ca’ Granda Ospedale Maggiore Policlinico, 20122 Milan, Italy; 5 Division of Hematology and Hemostaseology, Department of Medicine I, Medical University of Vienna, 1090 Vienna, Austria

**Keywords:** cancer, venous thromboembolism, sex, gender, hemostasis, inflammation

## Abstract

Cancer-associated thrombosis (CAT) is a major cause of morbidity and mortality in oncology, arising from complex interactions between tumor biology, host factors, and anticancer therapies. Growing evidence indicates that biological sex and gender-related factors modulate both thrombotic risk and clinical expression of venous thromboembolism (VTE) in patients with cancer. In this narrative review, we summarize current epidemiological, biological, and clinical data on sex- and gender-related differences in CAT across solid and hematologic malignancies. Men generally exhibit a higher overall incidence of VTE, whereas women may experience earlier, treatment-associated thrombotic events, with variability according to cancer type, stage, and therapy. Biological factors linked to coagulation and inflammation differ between sexes and may contribute to these patterns, although mechanistic evidence remains incomplete. Sex-related disparities also emerge in treatment-associated complications, including bleeding risk and abnormal uterine bleeding in anticoagulated women of reproductive age. In contrast, evidence for sex differences in oncohematology-associated thrombosis is limited and inconsistent. Gender-related inequalities in clinical trial participation further constrain the interpretation of available data. Overall, current evidence supports sex as a clinically relevant modifier of CAT risk, underscoring the need for systematic sex- and gender-informed research, to improve mechanistic understanding, and sex-stratified reporting to advance precision medicine in thrombosis and oncology.

## 1. Introduction

Cancer constitutes one of the leading causes of morbidity and mortality worldwide, imposing a substantial burden on public health systems and exerting profound physical, psychological, and socioeconomic impacts on affected individuals. Importantly, marked sex-based differences exist in cancer epidemiology, reflecting a complex interplay of biological, hormonal, genetic, and environmental factors that influence both cancer incidence and outcomes [1].

Venous thromboembolism (VTE), mainly including upper- or lower-extremity deep vein thrombosis (DVT) and pulmonary embolism (PE), is a common complication among patients with cancer [2], reaching up to 12-fold the risk of the general population [3,4]. Beyond this increased baseline risk, cancer is also associated with a greater likelihood of recurrence: patients with malignancy have approximately a 3-fold higher risk of recurrent VTE compared with individuals without cancer, and they are more prone to bleeding complications while receiving anticoagulant therapy [5]. Active cancer and ongoing antineoplastic therapy are recognized as key drivers of thrombotic risk, whereas renal impairment, advanced age, thrombocytopenia, and certain tumor locations are associated with an increased likelihood of bleeding during treatment [6]. In addition, the baseline risk of bleeding is increased even without anticoagulant therapy [7].

Evolutionarily preserved programs that coordinate inflammation and coagulation have been selected to confine tissue damage caused by infections and trauma, thereby limiting the dissemination of exogenous agents [8,9]. Under physiological conditions, the coordinated activation of inflammation and coagulation helps control tissue damage and prevent pathogens from spreading. However, in the neoplastic setting, cancer cells reuse these pathways for their own advantage, so that coagulation no longer results in containment of disease but rather in a prothrombotic and hypofibrinolytic microenvironment that favors tumor growth, survival, angiogenesis, and metastatic spread [10,11].

The main mechanisms through which cancer cells enhance the procoagulant activity of host vascular cells include the release of soluble mediators such as inflammatory cytokines (e.g., TNF-α, IL-1β), proangiogenic and growth-promoting factors such as vascular endothelial growth factor (VEGF), fibroblast growth factor (FGF), and granulocyte colony-stimulating factor (G-CSF), as well as platelet-activating agonists including adenosine diphosphate and thrombin; and the expression of surface adhesion molecules and/or receptors that enable tumor cells to recruit circulating blood cells and adhere to the vessel wall. The contribution of platelets is particularly relevant, as they help preserve vascular integrity within tumors. The increased platelet activation and aggregation correlate with the metastatic potential of cancer cells in both in vitro and in vivo models of experimental metastasis. Platelet aggregation protects tumor cell surface from immunological recognition in the circulation, as tumor-induced platelet aggregation (TICPA) results in a “platelet coating” of cancer cells shielding them from natural killer (NK) cells [10].

Many different factors may contribute to the risk of developing cancer-associated thrombosis (CAT), including patient-related variables such as biological sex, ethnicity, hospitalization, prolonged immobility, history of VTE, comorbidities, site and stage of cancer, chemotherapy and hormonal therapy [10,12].

In population-level studies, age-adjusted incidence rates of first VTE are generally higher in men than women after accounting for female reproductive risk factors, whereas in younger women reproductive factors such as pregnancy and combined hormonal contraceptive use markedly increase women’s VTE risk during the reproductive years.Specifically, hormonal contraceptive use and pregnancy are well-established VTE risk factors in women of childbearing age, and when these are accounted for, men exhibit a higher intrinsic VTE incidence [13,14]. Meta-analyses and large cohort studies of recurrent VTE are controversial, revealing that male sex is associated with a significantly higher risk of VTE recurrence compared with female sex, particularly after unprovoked VTE, while other studies demonstrate that female sex has higher risk of cancer-associated VTE overall [14,15]. The overall age-adjusted annual incidence rate of VTE is higher for men than for women, with somewhat higher rates in women during childbearing years (16–44 years) compared with men of similar age, and higher rates in men after 45 [16]. Large cohort data also indicate that women with early (<40 years) or late menopause (age > 55 years) have a significantly increased VTE risk, supporting menopause as a potential modifier of CAT susceptibility [17].

Postmenopausal hormone therapy, particularly oral estrogen, further increases VTE risk, although the magnitude of risk varies substantially according to formulation and route of administration. In particular, transdermal estrogen appears to have a more neutral thrombotic profile [18].

At the same time, sex differences in cancer-associated VTE recurrence and overall risk are less consistent, with several cohort analyses reporting similar recurrence rates in men and women with active cancer, and cancer-specific studies showing mixed outcomes by sex [19,20].

Gender Medicine is a rigorous medical approach that integrates biological sex and gender-related differences into the study, diagnosis and management of diseases. Its ultimate goal is to advance personalized precision medicine by enabling more accurate and effective treatments tailored to individual characteristics [21]. Here, we describe sex and gender differences in CAT, emphasizing how their consideration improves risk stratification and treatment selection, with the aim of advancing precision medicine.

## 2. Materials and Methods

In this narrative review, we conducted a comprehensive search of the English-language literature indexed in the Medline/PubMed database from January 2020 to October 2025. The search strategy included the following keywords, used individually and in combination: “cancer”, “thrombosis”, “cancer-associated thrombosis”, “sex”, and “gender”. We considered both original research articles and review papers that addressed epidemiological, mechanistic, or clinical aspects relevant to sex- and gender-related differences in CAT. Studies without accessible full text, conference abstracts, commentaries, and papers not pertaining to CAT were excluded. Reference lists of selected articles were also searched for additional relevant publications. Earlier seminal studies were included beyond 2019 when highly relevant.

This work is a narrative review and as such it may therefore be subject to selection bias and incomplete coverage of the available literature. The evidence summarized here reflects studies identified through targeted searches and expert knowledge. The heterogeneity of designs, endpoints, and reporting across the included studies further limits the ability to compare findings directly or quantify effects. These constraints are inherent to the narrative format and should be considered when interpreting the conclusions. Nonetheless, this approach allows integration of emerging evidence, clinical context, and expert insight in an area where long-term, high-quality data remain scarce.

## 3. Sex-Related Differences in Thrombotic Risk in Solid Tumors

CAT results from a multifaceted interaction between tumor-related, patient-related, and treatment-related determinants, many of which exhibit sex-specific variations that shape individual thrombotic risk profiles. Tumor characteristics (including cancer type, histological grade, and disease stage) are major predictors of CAT, with a particularly high incidence observed in pancreatic, gastric, lung, and gynecologic malignancies [2,12]. Patient-related features such as age, hormonal environment, adiposity, and baseline coagulation status differ between men and women and may lead to sex-dependent differences in susceptibility to CAT [13,22]. Furthermore, therapeutic exposures, such as hormonal therapy, cytotoxic chemotherapy, targeted agents, and oncologic surgery, can further modify thrombotic risk in a sex-dependent manner due to variations in drug metabolism, vascular reactivity, and inflammatory pathways [19,20].

### 3.1. Cancer Type

A subset of malignancies, several of which are among the most prevalent worldwide, develop in sex-specific organs, namely prostate cancer in men and uterine or ovarian cancers in women (Table 1) [23]. Sex hormones and tissue-specific susceptibility both play a strong role in carcinogenesis. As a consequence, also CAT incidence is different in the different types of sex-specific tumors. Breast cancer, although biologically possible in both sexes, remains exceedingly rare in males, with an estimated incidence of approximately 0.4 per 100,000 person-years, nearly 100-fold lower than that observed among females [24]. In breast cancer, the absolute VTE risk spikes around systemic therapy: population data suggest ~6% per year during chemotherapy and the month following end of treatment [25]. Endocrine therapy further modifies risk: tamoxifen roughly doubles the odds of VTE (adjusted OR 1.95, 95% CI 1.45–2.62), with a dose–response pattern [26], whereas aromatase inhibitors (AIs) carry a lower VTE hazard than tamoxifen (adjusted HR 0.59, 95% CI 0.43–0.81) [27]. Data on VTE in male breast cancer remain scarce, but available evidence suggests that thrombotic risk may be clinically relevant, particularly in the context of endocrine therapy. In a prospective cohort of 218 men with hormone receptor–positive breast cancer, tamoxifen exposure was associated with an increased incidence of thromboembolic events, with an estimated incidence of 51.9 per 1000 person-years compared with 21.5 per 1000 persons-years. Notably, most events occurred within the first 18 months of therapy [28]. In ovarian cancer, one of the most thrombogenic solid tumors, a Danish cohort reported a 2-year cumulative VTE incidence of ~7.2% [29], and a recent meta-analysis in advanced disease treated with neoadjuvant chemotherapy found a pooled VTE incidence of about 10%; obesity (BMI ≥ 30 kg/m^2^) increased the risk (pooled OR 1.76, 95% CI 1.13–2.76) [30]. For endometrial cancer, overall VTE incidence around diagnosis and treatment has been reported at ~6.2%, with postoperative 60-day VTE near 0.7% after surgery (higher with non-endometrioid histology, stage III–IV disease, and laparotomy) [31]. Even higher risk has been detected when chemotherapy is the primary modality compared with surgery or hormone therapy [32]. Together, these data show that hormone-responsive tumors and their treatments (Selective Estrogen Receptor Modulator—SERMs vs. AIs, systemic chemotherapy, and surgical approach) measurably shape CAT risk in women. Prostatic cancer has a reported incidence of CAT of 5–10/1000 per year and the use of androgen deprivation therapy is associated with a 2-fold increased risk [33].

Sex differences in solid tumors are observed in cancer type incidence, with males having higher rates in most cancers like bladder, colon, liver, and skin, while females have higher incidence in thyroid and gallbladder cancers (Table 2) [34,35]. Esophageal adenocarcinoma, laryngeal cancer, and gastric cardia cancer showed the strongest discrimination and were also characterized by the most pronounced male predominance after adjustment for demographic, lifestyle, and dietary factors. In contrast, men exhibited a lower risk of thyroid and gallbladder cancers compared with women. Overall, the increased relative risk among men persisted across multiple cancer types after adjustment, whereas for several sites including lung, pancreas, small intestine, colon, oral cavity, esophageal squamous cell carcinoma, and other head and neck cancers, the apparent male excess was largely attenuated after accounting for lifestyle and dietary variables. These differences are likely influenced by gender (behavioral and environmental factors), including variations in dietary patterns, alcohol consumption, and tobacco use, as well as potential biological sex-related differences in mucosal defense mechanisms and metabolic processing of carcinogens [35]. In a recent prospective cohort at the National Institutes of Health-AARP Diet and Health Study (1995–2011), (N = 171,274/122,826 M/F participants; age, 50–71 years, 17,951/8742 incident cancers in M/F), cancer incidence was lower in men than in women only for thyroid and gallbladder cancers [35]. For instance, thyroid cancer occurs significantly more frequently in females, a phenomenon that may be partly explained by sex-specific immune modulation and the higher prevalence of autoimmune thyroid disorders among women. This observation suggests a potential link between endocrine–immune interactions and female predisposition to certain malignancies [35].

At most other anatomic sites, the risks were higher in men than in women (adjusted HR range, 1.3–10.8), with the strongest increases for bladder (HR 3.3, 2.9–3.8), gastric (HR 3.5, 2.3–5.4), larynx (HR 3.5, 2.5–5.1), and esophageal cancer (HR, 10.8, 7.3–15.9) [35]. Males also face higher cancer mortality rates and often have later diagnoses than females. Differences also occur in cancer stage at diagnosis, tumor microenvironment (TME), and response to therapies. Males have higher mutational rate, and X chromosome has a mutation buffering effect [34]. Pan-cancer analyses reveal sex-specific differences in TME features like tumor mutational burden (TMB), immune cell infiltration, and stromal cell presence [34]. Sex-based differences have been noted in the patterns of cancer metastasis.

These disparities stem from a complex interplay of factors including sex chromosomes, hormones, the gut microbiome, and immune system differences [34]. Indeed, differences in X-linked gene expression and cellular mosaicism in females contribute to sex-related variations in cancer susceptibility. Cellular mosaicism resulting from X-chromosome inactivation (XCI) may provide a biological framework for the earlier treatment-associated VTE peaks observed in women. Random XCI generates two functionally distinct cell populations that differentially express X-linked genes involved in immune regulation, endothelial activation, and coagulation pathways. Skewed or unstable XCI patterns, which have been reported in autoimmune diseases, can promote clonal expansion of pro-inflammatory or pro-thrombotic immune subsets, potentially amplifying transient treatment-related perturbations in hemostatic balance. This mosaic immune landscape may increase susceptibility to early endothelial activation and thromboinflammatory responses after initiation of immunomodulatory therapies, before compensatory mechanisms develop. Experimental and clinical evidence suggests that X-linked immune genes escaping inactivation and sex-biased epigenetic regulation contribute to enhanced immune activation and vascular risk in females, supporting this hypothesis [36,37]. Androgens and estrogens then influence the immune system and regulate directly cell behavior, affecting cancer susceptibility and prognosis. Females often have a stronger immune microenvironment, potentially influencing immune-related cancer progression and therapy response, though results can be conflicting across different cancer types.

In sex-neutral, high-thrombotic-risk malignancies such as lung, gastrointestinal/colorectal, and pancreatic cancers, sex remains a modest but clinically meaningful modifier of CAT risk. Across multiple cohorts, men show a slightly higher baseline VTE risk than women (HR 1.2 to 1.3), consistent with general population trends [19,38]. There is evidence that women have greater platelet reactivity influenced by estrogen status, which may underlie subtle sex-related differences in CAT expression [39].

In lung cancer, population data indicate a 1-year cumulative VTE incidence of ~3% and a 2-year incidence near 3.4% [40], while registry analyses confirm substantial early risk during treatment, with sex differences attenuating after age and comorbidity adjustment [41]. Importantly, sex interacts with tumor biology to influence thrombotic risk: women more frequently develop EGFR-mutated adenocarcinomas, whereas men are more likely to present with KRAS- or ALK-driven tumors, which are associated with stronger inflammatory signaling and tissue-factor expression [40,41]. These biologic distinctions parallel epidemiologic findings that women tend to experience earlier, treatment-associated VTE peaks, while men demonstrate higher overall cumulative incidence during the disease course [38,40,41].

In colorectal cancer, large population studies report a 1-year VTE incidence of ~1.9% compared with 0.2% in matched controls (hazard ratio ≈ 8.9), with only modest or inconsistent sex-specific gaps after adjusting for comorbidity [42]. In pancreatic ductal adenocarcinoma, one of the most thrombogenic tumors, the Base Clinico-Biologique de l’Adénocarcinome Pancréatique [BACAP] study reported VTE incidences of 8.1% at 3 months and 19.2% at 12 months [43], a finding corroborated by a nationwide Japanese registry [44]. Although sex-stratified pancreatic ductal adenocarcinoma incidence data are limited, US mortality analyses show higher VTE-related deaths in men than in women (age-adjusted mortality 0.46 vs. 0.35 per 100,000 in women) [45].

In melanoma, available data indicate a clinically relevant burden of CAT but only weak, inconsistent signals for sex-specific differences. In the ICI era, a dedicated melanoma cohort showed that among patients treated with immune checkpoint inhibitors, the cumulative incidence of thromboembolism reached 9.3% at 6 months and 16.0% at 12 months, with higher VTE rates under combination versus single-agent ICI (16.7% vs. 5.0% at 6 months), yet sex was not identified as a major determinant of TE risk [46]. More recently, a melanoma-only study of 315 ICI-treated patients (42% women) found incidences of 5.1% VTE and 2.2% arterial thromboembolism over two years; in multivariable competing-risk models, female sex was not associated with higher TE risk, whereas prior VTE and higher BMI emerged as relevant predictors [47]. By contrast, in a smaller mixed-cancer immunotherapy cohort (229 patients, VTE incidence 7%), female sex and melanoma diagnosis were both independently associated with increased VTE risk, suggesting a possible interaction between sex and melanoma biology under ICI exposure that has not yet been confirmed in melanoma-specific datasets [48].

Finally, pregnancy-associated cancer (PAC), defined as malignancy diagnosed during gestation or within the first postpartum year, represents a rare but increasingly recognized clinical scenario, occurring in approximately 1 in 1000 pregnancies, with breast cancer, cervical cancer, melanoma, lymphoma, and thyroid cancer being the most frequent entities reported in population-based studies [49]. Beyond the oncologic and obstetric challenges, PAC carries a substantial thrombotic burden. Malignancy further enhances coagulation activation through tumor-cell expression of tissue factor (TF), release of procoagulant microparticles, systemic inflammation, endothelial dysfunction, and treatment-related vascular injury. The coexistence of these two prothrombotic conditions may therefore synergistically amplify the risk of VTE.

Large registry-based and population cohort studies have demonstrated that women with PAC experience a significantly higher incidence of VTE compared with pregnant women without cancer, with the highest relative risk observed during the postpartum period [50]. The risk appears particularly elevated in women with metastatic disease, hematologic malignancies, or those receiving chemotherapy during pregnancy.

Overall, these findings underscore that even in cancers not driven by sex hormones, sex influences CAT risk through differences in tumor biology, systemic inflammation, and treatment exposure, supporting the need for sex-aware thrombosis risk assessment in oncology.

### 3.2. Stage and Grade

Tumor burden, reflected by advanced stage and high histologic grade, is among the strongest predictors of CAT. Across tumor types, metastatic disease increases VTE risk approximately 4- to 7-fold compared with localized cancer [51]. In the Vienna Cancer and Thrombosis Study, patients with distant metastases had a 3.9-fold higher VTE risk (HR 3.9, 95% CI 2.6–5.8) than those with a localized disease, while regional lymph-node involvement conferred an intermediate risk [HR 2.2 (95% CI 1.4–3.3)] [3]. Evidence on tumor grade is limited, but high-grade and poorly differentiated tumors consistently show 2- to 3-fold higher VTE rates compared with low-grade lesions [12]. Sex further modifies this risk. Although large cohorts rarely report stage-by-sex interactions, male sex consistently emerges as an independent predictor of CAT after adjustment for stage and treatment. In a population-based Danish study of 499,000 cancer patients, men had a 20% higher VTE risk than women (adjusted HR 1.20, 95% CI 1.14–1.27), and metastatic disease tripled the risk regardless of sex [3]. Similarly, in the international Cancer-VTE Registry, male sex was associated with a higher cumulative 12-month VTE incidence (5.4% vs. 4.1% in women) after adjustment for age and cancer type [52].

Instead, women appear more prone to early-onset, treatment-associated events: in the PROTECHT trial sub-analysis, 70% of all VTEs in women occurred within 3 months of chemotherapy start versus 52% in men [53].

Collectively, these findings suggest that while high grade and aggressive biology universally heighten CAT risk, sex modulates the intensity and timing of this risk through hormonal and inflammatory pathways.

### 3.3. Treatment-Related Factors

Surgery remains a major trigger for CAT, with postoperative VTE risk peaking within the first 2–3 months after surgery and varying by tumor site, operative extent, and recovery duration. There could be a female predisposition to CAT after surgery, due to interacting biological and clinical factors. Also, women undergoing colorectal or gynecologic cancer surgery, experience slower mobilization and longer hospital stays, further extending the window of venous stasis and clot formation [54]. Across major oncologic procedures, a large U.S. Nationwide Inpatient Sample analysis (almost 2.5 million operations) found female sex independently associated with greater odds of in-hospital VTE after major cancer surgery (adjusted OR 1.25 vs. men) after multivariable adjustment [54]. In contrast, a prospective colorectal-cancer surgery cohort (2171 surgeries) reported no association between sex and 30-day symptomatic VTE after prophylaxis [55]. These results are consistent with a recent population study (432,218 cancer surgeries) that highlighted a marked time-dependent risk with HRs for PE 10 to 30 times higher at 30 days post-discharge depending on cancer type, but failed to demonstrate a consistent independent sex effect after adjustment [56].

Across oncologic therapies, both the intensity and toxicity of systemic treatment differ by sex, and these disparities may extend to thrombotic complications. A recent meta-analysis of 23,296 patients from 202 randomized trials found that women had a 34% higher odds of developing severe (grade ≥ 3) adverse events than men (OR 1.34, 95% CI 1.27–1.42; *p* < 0.001), with the gap most pronounced for immunotherapy (OR 1.49, 95% CI 1.24–1.78) [57]. But focusing on CAT risk during cytotoxic chemotherapy, prospective registry data show that male sex independently increases this risk during and after treatment: in 1583 ambulatory adults starting chemotherapy, men had a 57% higher hazard of VTE over 3 years than women (HR 1.57, 95% CI 1.21–2.07) [58]. Population-based analyses confirm a modest male excess in cancer-associated VTE during active systemic therapy (HR 1.20, 95% CI 1.14–1.27), independent of regimen or cancer stage [3]. For immune checkpoint inhibitors (ICIs), large multicenter cohorts report 6- to 12-month VTE incidences of 7–11%, but sex is not an independent predictor after adjustment for tumor type and inflammation markers—female sex occasionally associates with earlier events, yet differences disappear in multivariable models [58].

With targeted and anti-angiogenic agents, VTE risk is primarily drug-driven: bevacizumab raises an increased risk of VTE in patients administered with the drug compared with those not administered the drug (RR 1.33, 95% CI 1.13–1.56), while broader meta-analyses of tyrosine-kinase inhibitors show increased risk overall without a consistent sex interaction [59,60]. Overall, available data suggest that systemic therapy amplifies baseline sex differences in CAT, with men showing higher adjusted VTE hazards during chemotherapy, whereas for ICIs and targeted agents, sex-specific patterns remain uncertain.

In a single-center retrospective study conducted in cancer patients treated with anti-Programmed cell Death 1 (PD-1), anti-Programmed cell Death Ligand-1 (PD-L1), anti-Cytotoxic T-Lymphocyte-Associated Protein 4 (CTLA4), a combination of anti-PD-1/anti-PD-L1 and anti-CTLA4 or a combination including any of these drugs with chemotherapy, antiangiogenic agents or both the incidence of VTE was 7%, with female sex and melanoma correlating with an increased risk of VTE [48].

### 3.4. Device-Related Thrombosis

Central venous catheters and implanted ports are indispensable in modern oncology, allowing long-term administration of chemotherapy, parenteral nutrition, and supportive agents. However, these devices are a leading cause of upper-extremity venous thrombosis, contributing to approximately 20–30% of all cancer-associated thrombotic events [61]. The incidence of catheter-related thrombosis (CRT) varies by catheter material, insertion site and cancer type, ranging from 2% to 5% in solid tumors and up to 20% in hematologic malignancies [62].

Evidence regarding sex-based differences in CRT risk remains limited but points to possible anatomical and physiological contributors. Ultrasound and venographic studies show that women generally have smaller central venous diameters and thinner vessel walls, which may increase venous stasis and turbulent flow around catheters [63]. In contrast, men more often have higher baseline hematocrit and fibrinogen levels, promoting catheter-related fibrin sheath formation [64]. A meta-analysis similarly found that female sex, low BMI, and smaller vein–catheter ratios increase CRT risk, particularly when catheters are placed in the subclavian vein [65].

Altogether, current data suggest that while sex alone is not an independent determinant of CRT, anatomical and physiological differences may transiently increase CRT susceptibility in women.

Collectively, tumor, host, and treatment factors interact to create heterogeneous thrombosis risk patterns across sexes, highlighting the need for sex-informed risk assessment and personalized thromboprophylaxis strategies in oncology [66].

### 3.5. Biological Sex Factors

There are complex sex differences in CAT, however its overall risk depends on cancer type, treatment and age, which overall influence epidemiology [6]. Most of the available evidence in the field derives from registries including patients who were treated after developing a thrombotic event at the moment of cancer diagnosis [6,15,67], entailing some selection bias.

Epidemiologically, several studies have previously demonstrated that men generally exhibit a higher overall incidence of VTE compared with women [68,69], while recent studies conducted specifically on CAT have not consistently confirmed these findings [15,70].

Indeed, among 33,462 unselected patients with PE, this was less frequently caused by cancer in women than in men [71]. Most studies and three large independent registries reported that men generally experience a higher overall incidence of VTE, while women show more severe disease symptoms and recurrent VTE, but also lower death rates from CAT and overall survival [6,15,67]. However, a recent large study from a Swiss cohort of more than 1,000,000 patients showed a higher incidence of VTE in female cancer patients compared to males when affected by solid tumors, and an identical rate among patients with hematological malignancies [19].

These figures may be affected by cancer type and confounding factors, as among 1008 Japanese patients with lung cancer VTE was more common in women at 2-year follow-up [72]. The risk of bleeding after anticoagulation was differentially affected by the clinical features of the specific study cohorts, and more data is necessary to draw reliable conclusions [6,15,67]. Women appear more prone to CAT in some cohorts, but this pattern partly reflects underlying cancer-type distribution, as breast, hematologic and brain tumors confer intrinsically higher thrombosis risk. Treatment approaches also differ, with women sometimes receiving less aggressive therapies despite a higher severity of disease, although these patterns have not been fully explored in the context of CAT [6,15,67].

**Figure 1:**
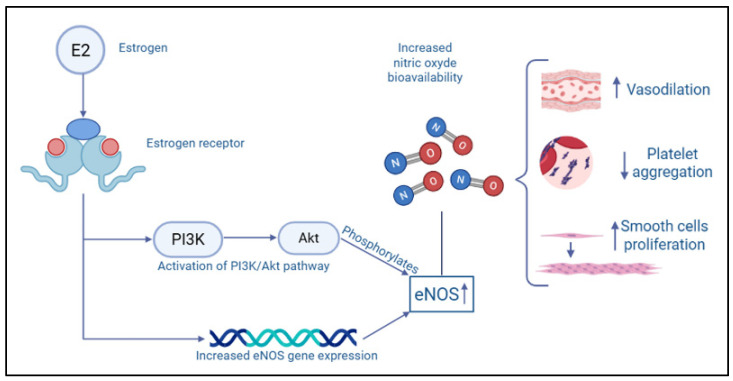
Effects of estrogen on endothelial nitric oxide synthase (eNOS) and nitric oxide availability. Akt = protein kinase B; E2 = Estrogen; eNOS = endothelial nitric oxide synthase; NO = nitric oxide; PI3K = Phosphoinositide 3-kinases; ↑ = increase; ↓ = decrease; → = leading to.

Sex-related biological factors implicated in CAT risk are still under investigation, and most mechanistic evidence remains indirect. Although direct sex-by-grade interaction analyses are rare, several biological pathways differ between men and women and may plausibly modify the thrombotic response to cancer. Males generally exhibit more pro-inflammatory innate responses in some contexts, which may enhance TF–FVIIa-mediated thrombin generation within the tumor microenvironment, thereby facilitating a procoagulant state [10]. In contrast, estrogen signaling in females modulates endothelial and platelet biology through multiple pathways: estradiol upregulates endothelial nitric oxide synthase (eNOS) activity, increases nitric oxide (NO) bioavailability and influences adhesion molecule expression, while also affecting platelet–vessel wall interactions and the balance between prostacyclin and thromboxane (Figure 1). These mechanisms may contribute to the earlier onset of treatment-associated thrombotic events reported in women [12].

Women exhibit higher baseline platelet reactivity and reduced responsiveness to platelet inhibition compared with men, a phenomenon attributed to sex differences in platelet intracellular calcium mobilization, surface receptor expression and downstream activation pathways [73]. Plasma levels of factors II, VII, VIII, IX, X, XI, and XII are higher in women than in men [74], favoring a hypercoagulable milieu that may be amplified by inflammatory states, trauma or surgery [75]. Data on clotting factor levels in women across different phases of the menstrual cycle are controversial: a recent study, however, found no significant differences in clotting factor concentrations between the follicular and luteal phase [76]. Hormonal exposure, rather than biological sex alone, contributes substantially to observed variations in coagulation profiles, highlighting the need to interpret sex differences within the context of menopausal status and ageing [77]. Oral estrogens increase VWF levels through stimulation of endothelial activity and enhanced platelet activation and adhesion. They promote thrombin generation and fibrin clot formation by upregulating hepatically synthesized coagulation factors, including FVII, FVIII, FX, FXIII, fibrinogen, and activated protein C. In parallel, oral estrogen reduces plasma concentrations of natural anticoagulant proteins such as TF pathway inhibitor, protein S, and antithrombin [78]. Because orally administered estrogen undergoes first-pass hepatic metabolism, these prothrombotic effects are largely mediated through liver-dependent pathways [79].

Available evidence suggests that sex differences in coagulation factors may diminish after menopause, as endogenous estrogen declines. Menopause leads to higher levels of fibrinogen, FVII, AT, and plasminogen [80]. FVIII is reported as higher [80] or unchanged after menopause [81]. However, VWF levels increase with age and should be taken into account for both sexes [82].

In addition, the contribution of iron deficiency (ID) whether absolute or functional in the context of cancer-related inflammation, can lead to thrombocytosis and increase the prothrombotic risk. This topic has not been extensively studied so far, especially with modern approaches, but several reports suggest that ID is associated with an increased risk of thrombotic events [83,84,85,86]. Iron restriction is common in cancer and can be exacerbated by chemotherapy, inflammation as a consequence of hepcidin-mediated iron sequestration. ID is associated with an increased risk of VTE possibly due to its role in immune system regulation and megakaryopoiesis, leading to secondary thrombocytosis with increased platelet reactivity and aggregation potential [87]. In vivo and translational work shows that ID can increase thrombus formation and a hypercoagulable milieu, and these changes can be mitigated by iron repletion [87,88]. Therefore, ID should be considered when evaluating patients at risk of CAT.

Emerging experimental and clinical data suggest that clonal hematopoiesis of indeterminate potential (CHIP) also contributes to prothrombotic and pro-inflammatory effects in cancer. Sex-related differences also exist in the epidemiology and genetic landscape of CHIP although their impact on thrombosis risk appears to be mutation-specific largely determined by the specific driver mutation, rather than uniformly sex-driven [89,90]. No sex-based differences emerge in the prevalence of CHIP, although men more frequently harbor *ASXL1* or *SRSF2* mutations, whereas women more frequently have *DNMT3A* mutations, which may be associated with distinct inflammatory profiles [91,92].

Neutrophil extracellular traps (NETs) are increasingly recognized as key mediators of CAT, as tumor-driven inflammation promotes NETosis, providing a procoagulant scaffold that enhances platelet adhesion, TF activity and thrombin generation [93,94]. Experimental and translational studies demonstrate that cancer-derived mediators prime neutrophils toward NET formation, contributing to thromboinflammatory pathways relevant to CAT. Emerging evidence suggests that NET formation may be influenced by sex hormones and neutrophil biology, with experimental data indicating differential NETosis responses between male and female neutrophils. In particular, in females, endogenous gender hormones may promote NETs during infection [95]. However, direct clinical evidence for sex-biased NETosis specifically in CAT populations remains limited. Unfortunately, scant data are available on the sex specific impact of genetic predisposition to thrombosis on the response to therapy. Cancer-specific studies indicate that inherited thrombophilia due to factor V (FV) Leiden and prothrombin G20210A are associated with a higher risk of VTE after cancer diagnosis, but most cohorts are not powered to test sex-by-genotype interactions and seldom report sex-stratified estimates; for instance, the Vienna Cancer and Thrombosis Study reported increased CAT risk associated with FV Leiden. Patients with FV Leiden and VTE were more frequently women with less frequent pulmonary embolism. However, due to the small number of patients with the event, additional statistical analyses of these variations could not be performed [96]. A recent systematic review/meta-analysis likewise supports FVL, prothrombin G20210A, and non-O blood type as genetic risk factors for CAT, while emphasizing that inherited thrombophilia (i.e., FVL and Prothrombin G20210A) is not common, with the consequence that achieving a sufficiently powered study would require testing for inherited thrombophilia in a large number of patients at considerable resource utilization [97].

Data on how inherited thrombophilia interacts with sex to influence CAT risk or response to anticoagulant therapy are extremely limited, and no adequately powered studies have addressed this question in cancer populations.

## 4. Sex-Related Differences in Thrombotic Risk in Oncohematology

When examining VTE in the context of oncohematology, the evidence for sex-related differences becomes considerably less consistent. In lymphoma, studies consistently identify aggressive histology, high tumor burden, and anthracycline-based chemotherapy as primary risk factors for thrombosis, with sex not emerging as a predictor in multivariable models [98]. In multiple myeloma, the introduction of immunomodulatory drugs (IMiDs) such as thalidomide and lenalidomide dramatically increased VTE incidence and sex does not significantly modify this risk [99]. Likewise, bleeding complications on anticoagulation do not appear to differ by sex in hematologic malignancies, with thrombocytopenia, mucosal involvement, and treatment intensity being far more influential [100]. The literature also highlights notable gaps: very few studies are designed to examine sex differences as a primary research question; data disaggregated by sex are inconsistently reported; finally, research on women of reproductive age with hematologic malignancies is particularly sparse [101]. In addition, it must be noted that most large studies or registries of cancer-associated bleeding do not stratify or report data specifically for hematologic malignancies only, but mix solid and hematologic cancers, so extrapolation is imperfect [102]. Therefore, future research with intentional sex-disaggregated analyses is warranted, given the increasing emphasis on personalized medicine and health equity.

## 5. Sex Differences in VTE Pattern

Even though biological sex is a known determinant of thrombotic risk in the general population, overall patterns of PE vs. DVT and distribution of sites are largely similar by sex, with only subtle differences in severity or presentation.

When the localization and type of thrombotic events are considered, available evidence remains discordant; however, overall patterns appear broadly similar between men and women. In particular, according to a Spanish study including 11,055 patients, the most common presentation of VTE was PE, accounting for 52% of the cohort for both sexes. Subtle differences were observed in the severity of clinical presentation, with women manifesting more hypotension, tachycardia and hypoxemia. In patients with initial DVT, proximal DVT was the most common presentation (71% for women, 72% for men), followed by upper limb DVT (14% versus 13%) [20]. In another study, derived from the same cohort and focusing on five different types of cancer (lung, colorectal, pancreatic, hematologic and gastric cancer), data showed that women with VTE were more likely to have colorectal, pancreatic or hematologic cancer than men, and less likely to have lung cancer and metastatic cancer. In this subgroup, which included 5135 patients, results showed a similar incidence of PE between men and women (54% and 53%, respectively), with no biological sex differences in the clinical signs of severity (arterial hypotension, tachycardia or hypoxemia). Also, no significant differences between men and women were observed based on site of DVT, with proximal DVT being the most common (64% and 67% respectively), followed by bilateral DVT (7.7% and 7.4%) and upper limb DVT (20% and 17%) [15].

Another international study, based on the GARFIELD-VTE database, including 10,650 patients, showed similar relative proportions of patients with primary diagnosis of DVT, PE, or DVT and PE, similar localization of DVT, balance of unilateral and bilateral DVT, and similar type of lower limb DVT [67].

In a study by Jara-Palomares et al., conducted on patients with a newly diagnosed VTE and occult cancer (followed for at least two years or until cancer diagnosis), no significant differences in the localization of DVT were found based on sex. However, when comparing cancer sites according to sex, men were more likely to have lung cancer than women and less likely to have pancreatic cancer. In this cohort, lung and colon cancer accounted for more than 50% of total occult cancer-related VTE in men, while in women there was a heterogeneity of cancer sites [66].

Finally, an analysis of 2823 patients from the international TESEO Registry demonstrated a significantly higher incidence of central venous catheter-related VTE in women than in men (27% vs. 21.4%). Among women, 71% of events were associated with Port-a-Cath devices and 28% with peripherally inserted central catheters, compared with 66% and 32%, respectively, in men. Of the 277 CRT, 26.0% (N = 72) occurred in patients with colorectal carcinoma. Breast cancer was the second most frequent tumor (22.4%, N = 62), despite a theoretically lower risk of thrombosis, likely because of a greater catheter use in this population. In both men and women, the most common VTE was PE (58.0% and 54.3%, respectively), followed by DVT (37.9% vs. 40.6%), with more than 9% of the subjects presenting simultaneously with PE and DVT in both groups. No significant differences were found between sexes with respect to the incidence of splanchnic thrombosis [6].

Beyond DVT and PE, cancer can also predispose to site-specific venous events such as portal vein thrombosis (PVT) in hepatocellular carcinoma (HCC), which similarly displays sex-related variation and is therefore relevant to mention.

The pathogenesis may be mediated both by portal hypertension (with reduction in flow velocity) and hypercoagulability, and by direct invasion of the portal branches by the cancer [103,104]. In 1752 Asian patients with HCC, PVT was a more frequent complication in men than in women [105].

## 6. Management of Cancer-Associated Thrombosis

The management of cancer-associated thrombosis relies primarily on anticoagulation, with low-molecular-weight heparin (LMWH) and direct oral anticoagulants (DOACs) representing the main therapeutic options.

Compared to men, women have been reported to experience 25% greater exposure to dabigatran and 15% greater exposure to apixaban, based on area under the curve (AUC) measurements. Moreover, female sex has been independently linked to approximately 1.2-fold higher plasma concentrations of apixaban [106]. By contrast, no significant sex-related differences have been observed in the pharmacokinetics of rivaroxaban or edoxaban. On the other hand, it is known how cytochrome P450 3A4 (CYP3A4) expression is higher in females compared to males [107]. However, to our current knowledge, there is no data supporting the existence of strictly “sex-specific” CYP polymorphisms, therefore observed differences are more likely attributable to sex-related differences in gene expression, hormonal regulation, and possibly differential allele frequency distributions across populations.

Early randomized trials established LMWH as superior to vitamin K antagonists (VKA) for preventing recurrent VTE in patients with cancer [108]. More recently, phase III studies have shown that factor Xa inhibitors are non-inferior to LMWH for efficacy, at the cost of a variable increase in bleeding in selected tumor types (gastro-intestinal and genitourinary tracts): edoxaban reduced recurrent VTE but increased major bleeding compared with dalteparin in Hokusai VTE Cancer [109], and rivaroxaban lowered recurrence with a higher rate of clinically relevant non-major bleeding in SELECT-D [110]. In contrast, apixaban showed similar or reduced bleeding with comparable efficacy versus dalteparin in ADAM VTE and Caravaggio clinical trial, supporting its use as a safer DOAC option in many patients with cancer [111,112]. Current guidelines therefore support the use of LMWH or factor Xa inhibitors in most patients with CAT, with the choice individualized according to bleeding risk, gastrointestinal tract involvement, drug–drug interactions and tumor type [113].

The recently published API-CAT trial, evaluating reduced-dose apixaban for extended anticoagulation in cancer-associated VTE, enrolled a balanced population of men and women but did not identify sex as an independent predictor of either efficacy or bleeding outcomes, and no sex-by-treatment interaction was observed [114].

Across these treatment strategies, clinical outcomes vary substantially due to cancer biology, treatment intensity, and organ function, while sex-specific differences in anticoagulant tolerability and bleeding remain insufficiently characterized. Within this framework, certain complications disproportionately affect women and warrant a dedicated consideration.

Given these sex-specific differences in bleeding risk, particularly in women of reproductive age, therapeutic management requires careful consideration. Heavy menstrual bleeding (HMB) represents a clinically relevant sex-specific complication of anticoagulation in reproductive-age women. Observational studies consistently report a higher prevalence of HMB in anticoagulated women compared with the general population, with factor Xa inhibitors (particularly rivaroxaban) associated with the highest rates [115,116,117,118,119,120,121]. Indeed, rivaroxaban use was associated with an increased risk of HMB compared to apixaban or warfarin, with a prevalence of 45%, 23% and 35%, respectively [121]. A documented history of HMB prior to DOAC initiation is strongly associated with HMB that requires intervention within six months from starting anticoagulation, independently of oral anticoagulant type [121]. Moreover, a high incidence of HMB (60%) with a negative impact on the quality of life has been reported in a recent study also for women without a history of HMB prior to DOAC initiation [122].

Nonetheless, a documented menstrual history is available only in a minority of women, definition of HMB lacks standardization, uterine bleeding is often reported as menstrual-related or not, making it difficult to estimate a true incidence rate of HMB.

## 7. Gender-Related Inequalities

Gender-related inequalities in access to clinical trials remain a persistent and multifaceted challenge in biomedical research. Despite a significant progress in recognizing the importance of diversity and inclusiveness, women and gender-diverse individuals continue to be underrepresented in many clinical studies. To understand the magnitude of the problem, it is notable that one of the most cited articles in epidemiology of CAT is based on a US Veteran database, which includes more than 420,000 men and only 14,000 women. In this article, authors acknowledged that the lack of association between male sex and VTE risk might be partly explained by the small number of female patients enrolled [66].

Historically, women were often excluded from clinical research due to safety considerations about reproductive potential, perceived risk during pregnancy and higher fluctuation of hormonal levels which impact different biological systems. However, these practices have led to an imbalance in the generation of scientific evidence, resulting in a scientific literature that often reflects a male-biased view of disease mechanisms, treatment responses, and adverse effects. Sex and gender disparities in clinical trial enrollment continue to pose a significant challenge, despite advancements in the knowledge and dissemination of gender medicine [123]. Women continue to be underrepresented in clinical trials designed to assess the safety and effectiveness of drugs, despite the fact that sex and gender differences should be taken into account from study design to data collection and analysis [124]. Since their introduction in 2016, the sex and gender equity in research (SAGER) guidelines help researchers to design studies which can appropriately consider sex and gender differences; nonetheless, these guidelines have not been consistently implemented [125]. Clinical studies in acute care rarely include sex- and gender-based analyses or provide only data disaggregated by race and ethnicity, and gender related variables are often under-investigated. Finally, differences in cancer stage at diagnosis may contribute to apparent sex disparities. Women often undergo closer medical follow-up and screening, leading to earlier detection and potential diagnostic bias that can influence outcome measures, including thrombosis incidence. Therefore, analyses of gender differences should adjust for screening uptake and healthcare utilization (gender) to avoid misattributing epidemiological variations to intrinsic sex-related risk [21].

Recent policy initiatives have sought to address these inequities. Scientific societies and research institutions have increasingly addressed the issue of gender equality, publishing guidelines and recommendations for the integration of sex and gender-based analysis into preclinical, epidemiological and clinical research protocols and studies [126,127,128]. Also, regulatory agencies such as the U.S. Food and Drug Administration (FDA) and the European Medicines Agency (EMA) now encourage or require sex-based reporting and analysis in clinical trials. However, these measures often fall short in addressing the complexity of the gender spectrum due to a mostly binary classification of sex and gender, thus reducing the individual’s likelihood of being represented in research. Transgender and gender-diverse individuals are almost entirely absent from current CAT studies, and the thrombotic implications of gender-affirming hormonal therapy remain poorly characterized. This gap further highlights the need for clinical research that systematically incorporates gender-related variables and ensures the inclusion of populations receiving exogenous estrogen or androgen therapy.

To advance gender equity in clinical research, a multidimensional approach is required. Integrating sex and gender considerations at every stage of the research continuum, from hypothesis generation to data interpretation, should become standard scientific practice. Finally, fostering gender diversity within research teams and leadership positions can promote a more inclusive scientific culture, capable of tackling and solving gender inequalities in scientific studies.

## 8. Research Agenda

Current evidence supports the relevance of sex as a modifier of thrombotic risk in cancer, underscoring the importance of integrating sex-specific considerations into the prevention and management of CAT. Nevertheless, available data remains heterogeneous and incomplete (Table 3). As a consequence, this review has some limitations. Certain cohorts included in the available literature, such as veteran populations, are inherently sex-skewed and may limit the generalizability of findings. Moreover, the scarcity of sex- and gender-disaggregated analyses constrains the interpretation of differential risk profiles. To provide a comprehensive overview of CAT, high-quality studies were included even when one sex was predominantly represented, which may introduce bias and reduce the precision of sex-specific estimates. Overall, these considerations further highlight the need for future studies specifically designed to address sex and gender differences in CAT.

Future research should clarify how sex and gender interact with tumor biology, treatment exposure and comorbidities to influence the timing and magnitude of thrombotic risk. Most data come from heterogeneous cohorts or post hoc analyses, and adequately powered prospective studies are still lacking. Disease-specific cohorts, particularly in solid tumors with marked sex differences in incidence and treatment, are needed to evaluate how hormonal milieu, inflammatory activity, tumor microenvironment features and stage contribute to CAT expression in women and men.

A better understanding of biological mechanisms is also required. Sex hormones, sex-chromosome–linked pathways and immune modulation may all influence endothelial activation and coagulation, but these interactions remain poorly defined. Integrated approaches combining clinical variables with molecular and microenvironmental profiling may help identify sex-specific modifiers of thrombotic risk.

Interventional studies should incorporate sex-stratified analyses from the design phase, with sufficient power to evaluate differences in efficacy, bleeding risk and treatment interruption. In parallel, coordinated international registries with harmonized data collection would provide a more accurate picture of CAT in real-world settings. Such registries should also support post-marketing surveillance of anticoagulants used in oncology, allowing sex-specific assessment of effectiveness, safety, dose modification and recurrence. Given the high prevalence of abnormal uterine bleeding in anticoagulated women of reproductive age, additional data on its incidence, biological determinants and patient-specific risk factors are also needed to guide safer therapeutic choices and to optimize concurrent management. This data will be essential to refine risk prediction and to address personalized management strategies.

## 9. Conclusions

Current evidence supports the relevance of sex as a modifier of thrombotic risk in cancer, highlighting the need to integrate sex-specific considerations into prevention and management of CAT. A more systematic inclusion of sex and gender variables in translational studies, prospective cohorts and interventional trials is essential.

A deeper understanding of sex-based disparities in CAT will ultimately allow more accurate risk prediction and truly individualized strategies for prevention, early detection and treatment in oncology.

## Figures and Tables

**Table 1:** Incidence of sex-specific cancers.

Site	Incidence (Rate/100,000; 95% CI)	% of All Cancers
Male		
Testicles	1.1 (1.1–1.2)	0.4
Prostate	15.4 (14.1–16.3)	5.6
Female		
Ovary	3.5 (3.1–3.8)	1.2
Uterus	5.4 (4.9–5.9)	1.9
Breast	24.6 (22.9–26.3)	8.9

Adapted from Ref. [23]. CI = confidence interval.

**Table 2:** Incidence of sex-neutral cancers.

Type of Cancer	Incidence in Males (Rate/100,000)	Incidence in Females (Rate/100,000)	M/F Age-Adjusted Incidence Ratio (95% CI)
Female preference			
Thyroid cancer	11.3	19.6	0.59 (0.49–0.70)
Gallbladder cancer	2.5	3.9	0.65 (0.44–0.94)
Male preference			
Esophageal adenocarcinoma	22.4	1.8	12.19 (8.32–17.86)
Gastric Cardia	14.3	2.9	4.93 (3.59–6.77)
Larynx cancer	18	4.5	3.99 (3.07–5.17)
Bladder	84.6	21.2	3.96 (3.52–4.46)
Liver cancer	20.6	8.7	2.38 (1.96–2.89)
Lung cancer	249.9	211.2	1.18 (1.10–1.23)
Pancreas	48.8	41.3	1.26 (1.14–1.39)
Small intestine	7.0	5.5	1.28 (0.98–1.68)
Colon cancer	130.0	100.2	1.29 (1.21–1.37)
Oral cavity	13.6	9.1	1.50 (1.22–1.85)
Skin cancer	116.2	53.1	2.20 (2.03–2.38)
Esophagus squamous cell carcinoma	5.1	3.0	1.67 (1.18–2.37)

Modified from Ref. [35]. CI = confidence interval, M = male, F = female.

**Table 3:** Sex differences in Cancer-Associated Thrombosis (CAT).

	Women	Men
**Epidemiology (general VTE)**	Historically lower overall VTE incidence; some recent CAT-specific cohorts show equal or higher incidence in solid tumors [14,15,17].	Generally higher overall VTE incidence across registries [16].
**Clinical severity & recurrence**	More severe symptoms and recurrent VTE in some registries; lower CAT-related mortality reported in some cohorts [6,15,65].	Higher overall incidence; mortality differences inconsistent [14,15].
**Inflammatory profile**	Estrogen modulates endothelial function (↑ eNOS, ↑ NO), affects platelet–vessel wall interaction and prostacyclin/thromboxane balance [12].	Higher innate immune response and tissue factor levels → procoagulant tumor microenvironment [10].
**Platelet biology**	Higher baseline platelet reactivity; reduced response to platelet inhibition [39].	Lower platelet reactivity compared with women [39].
**Coagulation factors**	Higher plasma levels of factors II, VII, VIII, IX, X, XI, XII → hypercoagulable milieu [64,72].	Lower levels compared to women [64,72].
**Iron deficiency impact**	Iron deficiency–related thrombocytosis may amplify prothrombotic risk (data limited) [72,82,83].	Same mechanism possible; less specifically described [72,82,83].
**Genetic thrombophilia interaction**	Data extremely limited; no adequately powered studies.	Data extremely limited; no adequately powered studies.
**Time-dependent postoperative risk**	Increased PE risk within 30 days post-discharge, no independent sex effect after adjustment [54].	No consistent independent sex effect after adjustment [54].
**Chemotherapy-associated CAT**	Lower adjusted VTE hazard compared with men [57,58].	Higher VTE hazard during and after chemotherapy [57,58].
**PE vs DVT proportion**	Similar distribution (PE 52–58%) [15,20].	Similar distribution (PE 52–58%) [15,20].
**Severity of PE presentation**	In some cohorts: more frequent hypotension, tachycardia, hypoxemia; other cohorts show no difference [20].	Generally similar severity [20].
**DVT site**	Proximal DVT slightly most common (64–71%); similar rates of upper-limb and bilateral DVT [15].	Similar distribution [15].
**Catheter-related thrombosis (CRT)**	Higher incidence (27%); more Port-a-Cath–associated events [6]. Possibly increased susceptibility due to smaller venous diameters and vein–catheter ratio; female sex identified as risk factor in meta-analysis (especially subclavian placement) [61,62,63].	Lower incidence compared to women (21.4%) [6]. Higher hematocrit/fibrinogen may promote fibrin sheath formation [62].

CAT = cancer-associated thrombosis; CRT = catheter-related thrombosis; DVT = deep vein thrombosis; eNOS = endothelial nitric oxide synthase; NO = nitric oxide; PE = pulmonary embolism; VTE = venous thromboembolism; ↑ = increase; → = leading to.

## Data Availability

No new data were created or analyzed in this study. Data sharing is not applicable to this article.
